# Interdisciplinary Aspects of Childhood Obesity and Physical Fitness

**DOI:** 10.1155/2013/828463

**Published:** 2013-06-03

**Authors:** Jana Pařízková, Françoise Rovillé-Sausse, Denes Molnár

**Affiliations:** ^1^Obesity Management Centre, Institute of Endocrinology, Národní 8, 11694 Prague 1, Czech Republic; ^2^Museum National d'Histoire Naturelle, HNS-CP 135, 57 rue Cuvier, 75231 Paris Cedex 05, France; ^3^Department of Pediatrics, University of Pécs, Jószef A. u. 7. Pécs, H-7623, Hungary

During recent years, the increasing prevalence of obesity has manifested in earlier periods of growth, which results in much greater health risks in later life [1, 2]. Moreover, this problem has been arising in an increasing number of countries including those which are in economic transition or are in certain social strata in developing countries which have been introducing a typical way of life in industrially developed countries, that is, reduced physical activity and inadequate dietary intake, for all age categories all round the world [1]. However, this is riskier for growing subjects, especially during certain critical periods of development, when the consequences of the adaptation to undesirable life conditions can have much more negative consequences than during later life [3, 4]. 

Recent comparative studies during growth revealed not only increasing adiposity [5] in the general population but also a reduction of the physical fitness level in preschool [6] and adolescent school children [7, 8], which appeared along with increasing prevalence of overweight and obesity [1]. This is related to a prevailing reduction of physical activity and exercise. Psychologists comment that “a child can learn to learn, or learn *not* to learn”; it may be added that “a child can learn to move, or to learn *not* to move.” Early adaptation to increased physical activity can have a significant impact not only in the present health condition but especially in relationship to delayed consequences concerning physical fitness in later life [2, 9, 10]. The optimal level is a desirable component of health status, which should mean not only the absence of diseases but the state of full physical, mental, and social well-being [1]. 

 To have significant results due to the adaptation to work load and exercise, a certain character, intensity, duration, and frequency of variables have to be applied. When this is not adhered to, the results might not appear or be minimal—also with regard to adiposity and physical fitness level [3, 4, 9]. Mentioned characteristics of exercise have not always been available in studies that followed scientific protocols, and conclusions of an insignificant impact from physical activity and exercise in the prevention and thus the treatment of the obese have sometimes been lacking. 

Increasing experimental data concerning physical fitness and performance of the obese—especially in younger children—have been available more recently [3, 4]. It has been always difficult to assemble a group of obese children and/or adolescents sufficiently homogenous with regard to age, sex, and also duration and degree of obesity, comparable level of sexual development (which can also significantly change adiposity), and conduct adequate tests for physical fitness level. Many studies revealed reduced physical activity of obese children and adolescents, which could be a primary reason of excess adiposity, but also a consequence of it [3, 4, 7, 8]. 

Tread-mill, bicycle ergometer testing, and so forth have been always straining and unpleasant for the obese, and those without health complications are difficult to get in sufficient numbers. Generally, cardiorespiratory efficiency during transferring one's own body weight along a distance is mostly tackled by overweight and obesity [4]. Previous measurements revealed, nevertheless, sometimes quite high absolute values, for example, of oxygen uptake during maximal work loads [11]. However, when related to total measurements and especially lean body weight, mostly reduced values were found [4, 10, 12]. Moreover, a certain level of the maximal oxygen consumption during tread-mill or bicycle tests was achieved after a shorter testing periods. Obese subjects had to finish the dynamic work load earlier than normal weight subjects; that is, their ability to achieve maximal performance with the same oxygen uptake was significantly reduced [4]. After the reduction of treatment by exercise, along with the reduction of fat and BMI, the cardiorespiratory efficiency was also significantly increased and higher performance was achieved; that is, subjects were able to run for a longer time, with higher speed, and so forth than before treatment [10, 12]. But it should be mentioned that the ability of smaller muscle groups can be unchanged by obesity, and muscle strength is even increased due to enhanced development of lean (mostly muscle) body mass, especially in subjects with longer lasting excess adiposity development [4, 10]. 

The positive role of physical activity on obesity prevention and treatment has been previously revealed, but indispensably under conditions of a particular character (preferably aerobic, dynamic), intensity, duration, and frequency of exercise on fitness development, [4, 12]. Present life styles especially in larger urban agglomeration, leisure time activities, mainly sedentary, but very attractive (TV, video, etc.), have been the greatest problem. To realize a recommended regime of physical activity [13] has become difficult, usually nearly impossible (safety reasons, reduced availability of supervised areas for play, etc.). Support and promotion of such regimes require increased cooperation of pedagogic, communal, political institutions, and so forth [13, 14]. Simultaneously, this also concerns a significant promotion of adequate nutrition, which has been negatively influenced by the global marketing and advertisement of undesirable industrialized foods and beverages resulting in unhealthy food habits and dietary intake, moreover in disproportion to present needs and energy output. Therefore, not only educational and pedagogic impacts are needed starting with early childhood [14], resulting in optimal physical activity habits and fitness, but also environmental and social influences favouring desirable lifestyles must be promoted as well. This requires additional and enhanced effort to include innovative intervention programs with support and cooperation of governmental, educational, social institutions, and so forth. Without well-concerted and complex endeavor, it would be difficult to achieve efficient obesity prevention and treatment *using natural and physiological factors like physical activity and fitness development*, and not pharmacological, or gastric binding and so forth, approaches, which are also more costly. The presented issue should at least partly contribute to that.

*Jana Pařízková* *Jana Pařízková* *Françoise Rovillé-Sausse* *Françoise Rovillé-Sausse* *Denes Molnár* *Denes Molnár*
